# Abrasion arthroplasty increases mesenchymal stem cell content of postoperative joint effusions

**DOI:** 10.1186/s12891-015-0705-0

**Published:** 2015-09-12

**Authors:** Rainer Beckmann, Sebastian Lippross, Claudia Hartz, Mersedeh Tohidnezhad, Mónica S. Ventura Ferreira, Sabine Neuss-Stein, Andreas Seekamp, Sven Nebelung, Nisreen Kweider, Björn Rath, Holger Jahr, Thomas Pufe, Deike J. Varoga

**Affiliations:** Department of Anatomy and Cell Biology, RWTH Aachen University, Wendlingweg 2, 52074 Aachen, Germany; Department of Trauma, University Hospital of Kiel, Kiel, Germany; Department of Trauma and Orthopaedics, University Hospital Heidelberg, Bergheim, Germany; Department of Hematology, Oncology and Stem Cell Transplantation, Medical Faculty, RWTH Aachen, Aachen, Germany; Department of Orthopaedic Surgery, RWTH Aachen University, Aachen, Germany

## Abstract

**Background:**

Abrasion arthroplasty (AAP) is a procedure by which intrinsic cartilage healing is believed to be stimulated. Although clinically accepted for degenerative and traumatic cartilage lesions scientific evidence at a molecular level that proves the effect of AAP is scarce.

**Method:**

Mononuclear cells were extracted from postoperative joint effusions 21.5 h post AAP and simple debridement of cartilage lesions. Luminex, ELISA and FACS experiments were performed. Immunohistochemical stainings of cell cultures for cartilage markers were used to confirm the findings.

**Results:**

Postoperative joint effusions after AAP showed increased contents of Mononuclear cells compared to Arthroscopic Chondroplasty (ACP). BMP-4 and IGF were increased in AAP as complared to ACP. Mononuclear cells isolated after AAP express the MSC markers CD 73, CD 105, CD 90, CD 44 and are CD34 negative. Chondrogenic differentiation was demonstrated by positive staining for Sox9, collagen II, proteoglycan, chondroitin-4-sulfate.

**Conclusion:**

Our results support the clinical application of AAP as a procedure that enhances cartilage repair as an alternative to far more complex procedures that have gained popularity. Furthermore the data presented supports clinical investigations that recommend not to use suction drainage as by this procedure a considerable amount of the regeneratory potential of postoperative joint effusions might be extracted.

## Background

Cartilage defects lack intrinsic healing potential and therefore cause an enormous number of orthopedic interventions [1]. So-called “marrow stimulating techniques” i.e. abrasion arthroplasty and microfracturing are common practice but the clinical outcome varies considerably [2, 3]. Alternative modern methods i.e. autologous chondrocyte transplantation, stem cell augmented repair techniques and osteochondral transfer have gained popularity and seem to provide a fair clinical outcome but such procedures are technically demanding and require a highly specialized infrastructure. Finally all procedures requiring ex-vivo cell culture are time consuming, involve repeated surgical intervention and are costly.

Therefore, many surgeons still advocate abrasion arthroplasty (AAP) as a first-line treatment for cartilage defects of the knee joint as it is easy to perform and may be combined with other interventions like repair of meniscal lesions or correction of the limb axis. Nevertheless, the general notion seems that AAP is palliative, predominantly used for patients seeking an alternative to total knee replacement [4].

Based on follow-up studies using magnetic resonance imaging (MRI) and animal experiments, it is well accepted that debridement of osteoarthritic lesions via arthroscopy does lead to fibrous tissue formation at the site of the defect but the regenerative cartilage substitute is of inferior quality and prone to degenerate over time [5–7]. Several scientific reports have highlighted the importance of stem cells from synovium, periosteum or bone marrow for the regeneration of cartilage and therefore many modern techniques involve transfer of autologous mesenchymal stem cells (MSC) or periosteal cells into the defect [8–10]. MSC have particularly raised interest as they seem to adhere to cartilage lesions [11]. Despite the increasing evidence that the local delivery of MSC is of major importance for cartilage repair, there are no clinical studies that clarify whether relatively simple and commonly used surgical procedures like AAP are cause for the release of MSC into the joint cavity. We have therefore investigated into the cellular composition of postoperative joint effusion after AAP with regard to its MSC content.

## Methods

The study was approved by the institutional review board of the University Hospital Schleswig-Holstein Campus Kiel (Ethik-Kommission der Medizinischen Fakultät der Christian-Albrechts-Universität zu Kiel). Written informed consent was obtained from all patients.

### Study design

We studied 2 cohorts of adult caucasian patients who had only one joint affected by osteoathritis. Patients enrolled in this study underwent abrasion arthroplasty (AAP) *n* = 6–37 or abrasion chondroplasty (ACP) *n* = 6–21. The indication was assessed by MRT. The outerbridge score were assessed athroscopicly and documented by photographs at the beginning of the operation. The mean age of the patients who underwent AAP was 54.9 ± 12.1 years with an outerbridge score between I and III. The mean age of the patients of the ACP group was 41.6 years ±16.5 with an outerbrige score between III-IV.

### Cell isolation from joint effusions

The joint effusions were collected for 21 h ± 2.5 h post-operation. Patient consent was obtained according to the requirements of the University Hospital Schleswig-Holstein Campus Kiel. Undiluted joint effusion was used to isolate mononuclear cells by ficoll density gradient centrifugation (1.100 g/ml, Biochchrom AG, Berlin). The cells were cultured in DMEM high Glucose, 10 % FCS, 1 % Penincilin-Streptomicin, 0.1 mM MEM non essential amino acids, 1 % HEPES-buffer (Gibcco, Germany) in tissue flasks with standard surface for adherent cells (Sarstedt, Nümbrecht, Germany) at 37 °C and 5 % CO_2_. Non-adherent cells were removed by changing the medium after 3 days. The cells were expanded up to 21 days for further analysis.

### Cell counting

Cells were stained by trypan blue and living cells were counted with a Neubauer chamber.

### High-density culture

Only cells isolated after AAP could be extended in a sufficient manor to cultivate in high-density cultre. Therefor 300,000 cells where centrifuged at 250 g for 10 min in a 15-ml-polypropylene tube and further cultivated for 3 weeks at 37 °C and 5 % CO_2_ in the previously mentioned culture medium on a total volume of 10 ml. The Medium was changed every 2–3 days.

### Immunohistochemistry

For immunohistochemical analysis the cell pellets were fixed in 4 % neutrally bufferd formalin for 2 h and embedded in paraffin at 56 °C. Imunohistochemical stainings were performed on serial sections of 5 μm thickness with antibodies against Sox9 (pretreatment: heat retrieval 30 min in 11 mM citronic acid, pH 6, dilution 1:500; BS1597, Bioworld, USA), chondrogen matrix proteins: collagen type II (pretreatment: proteinase K (10 ug/ml) for 10 min, dilution 1:50; BT21-5000-60, biotrend, Germany), proteoglycan (pretreatment: Incubation in reduction-solution for 2 h at 37 °C (50 mM Tris, 200 mM NaCl, 10 mM DTT pH7.35) followed by incubation for 1 h at 37 °C in alcylation solution (40 mM iodoacetamide in PBS), dilution 1:20; 12/12/1-C-6, DSHB, USA) and chondroitin-4-sulfate (pretreatment: heat retrivial in 11 mM citronic acid, pH6 for 20 min, dilution1:200; MAB2030, Chemicon international, Germany). Binding of species-specific biotinylated secondary antibodies was visualized with AEC-substrate chromogen (AEC-Kit) (00-2007, Invitrogen, Germany). Sections were counterstained with hematoxylin.

### Luminex-assay/ELISA

Serum from postoperative joint effusions was prepared from postoperative joint effusions by centrifuging at 1760 g for 10 min. The supernatant was stored at −20 °C until analysis. IGF-1 analysis was performed by Luminex-assay (Millipore, Germany). BMP4, BMP7 and TGFβ contents were measured by ELISA (R&D-Systems, Germany). Analyses were performed of serum according to the manufacturer protocols.

### Flow cytometry

After a 21 day expansion period of mononuclear cells isolated from joint effusions the expression of CD34 (120-000-803, Miltenyi Biotec, Germany), CD44, (H4C4, DSHB, USA), CD73 (12-0739, eBioscience, USA), CD90 (555593, BD Pharmingen, USA) and CD105 (555690, BD Pharmingen, USA) was examined using flow cytometry. 250 000 cells per antibody staining were used. Cells were washed in PBS before being recovered from cultures and resuspended in FACS-buffer consisting of 1 % BSA in PBS. Cells were stained for 30 min at 4 °C using primary antibodies 1:100 diluted in FACS-buffer. Subsequently cells were washed twice with FACS-buffer prior to the incubation with 1:200 diluted secondary antibody FITC goal anti-mouse IgG/IgM (555988, BD Pharmingen, USA) at 4 °C for 30 min. After staining the cells were washed twice and analyzed using FACS Canto™ II flow cytometer running on FACSDiva software (Becton Dickinson, USA). Data were evaluated using FlowJo Software (Tree Star Inc., USA). Appropriate controls were included for the analysis.

### Statistical analysis

Statistical analysis were performed with Kruskal-Wallis-Test followed by Dunns post hoc testing and student’s *t*-test. Group differences were considered significant if p <0.05 (*), p ≤0.005 (**), p ≤0.001 (***).

## Results

**Fig. 1:**
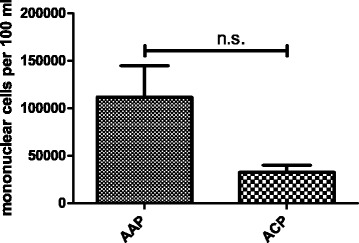
Mononuclear cells released after AAP and ACP. Postoperative joint effusion was collected for 21 h ± 2.5 h and mononuclear cells were isolated by ficoll density gradient centrifugation. Cells were counted after 1 week (*n* ≥ 8; mean, SEM)

First we assessed the contents of mononuclear cells in postoperative joint effusions 21 h ± 2.5 h post operatively. Effusions from patients undergoing AAP had a higher number of mononuclear cells than controls from chondroplasty alone (mean ± SEM: 111,923 ± 33,038 for AAP:, 32,500 ± 7460 for ACP; *n* ≤ 7, *p* < 0.0504) (Fig. 1).

**Fig. 2:**
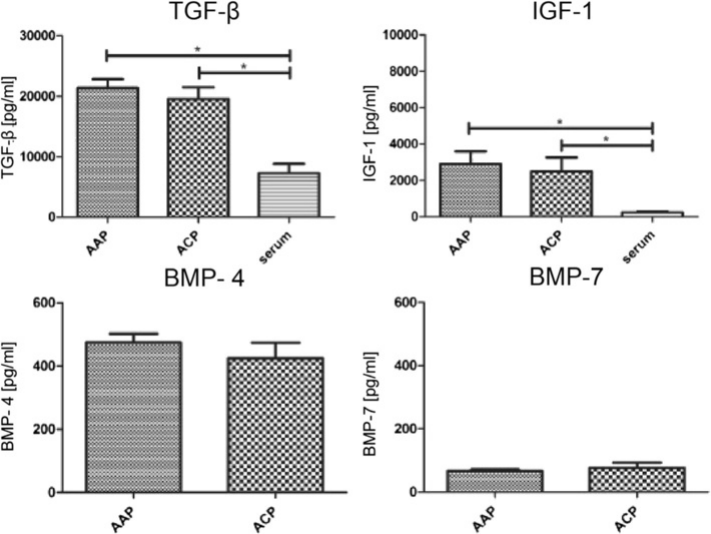
Chondrogenic growth factors TGF-β, IGF-1, BMP-4 and BMP7 in postoperative joint effusion released after AAP and ACP (Kruskal-Wallis-Test * *p* ≤ 0.05, ** *p* ≤ 0.005, *** *p* ≤ 0.001, *n* ≥ 6; mean, SEM)

In addition, the growth factor content of the cell-free fraction of joint effusions was analyzed by ELISA/Luminex to clarify whether the AAP provides a chondrogenic environment to promote cartilage healing (Fig. 2). The analysis of AAP and ACP revealed significantly higher concentrations of Insulin-like growth factor (IGF) (P value: <0.0001 AAP: 2902 ± 682 pg/ml, p value vs serum: ≤ 0.001, ACP: 2486 ± 759 pg/ml, p value vs serum: ≤ 0.001 Serum: 237 ± 40 pg/ml) and Transforming growth factor beta (TGF-β) (P value: <0.0001 AAP: 21,353 ± 1471 pg/ml p value vs serum: ≤ 0.001, ACP: 19,541 ± 1941 pg/ml, p value vs serum: ≤ 0.005 Serum: 7254 ± 1593 pg/ml) as compared to serum levels. Bone morphogenic protein 4 and BMP 7 were detectable in ACP and AAP but the BMP4 level was much higher than BMP 7 level (BMP-4 and BMP-7) (BMP-4: AAP: 474 ± 28 pg/ml, ACP: 425 ± 49 pg/ml; BMP-7: AAP: 66 ± 5.9 pg/ml, ACP: 77 ± 16 pg/ml). Although there were no significant differences between AAP and ACP, there was a trend towards higher concentrations after AAP in BMP 4 level.

**Fig. 3:**
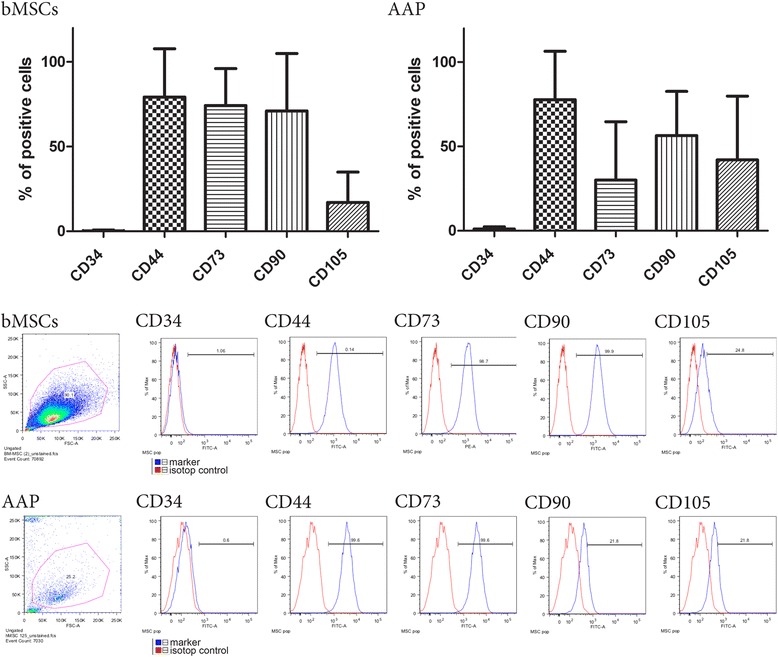
Mononuclear cells isolated after AAP express the MSC markers CD 73, CD 105, CD 90, CD 44 and are CD34 negative (MSCs *n* = 4, AAP *n* = 6)

In order to characterize the phenotype of the cells released after AAP and ACP a flow cytometry analysis was performed. Bone marrow (BM) derived-MSCs were analyzed within passages 3 to 6 for comparison. Remarkably, AAP-released cells show a CD73^+^, CD105^+^, CD90^+^, CD44^+^, CD34^−^ expression pattern (Fig. 3) coinciding with the typical MSC pattern defined by the criteria of the International Society for Cellular Therapy [12].

ACP -released cells could not be expanded to a sufficient number for flow cytometry analysis.

**Fig. 4:**
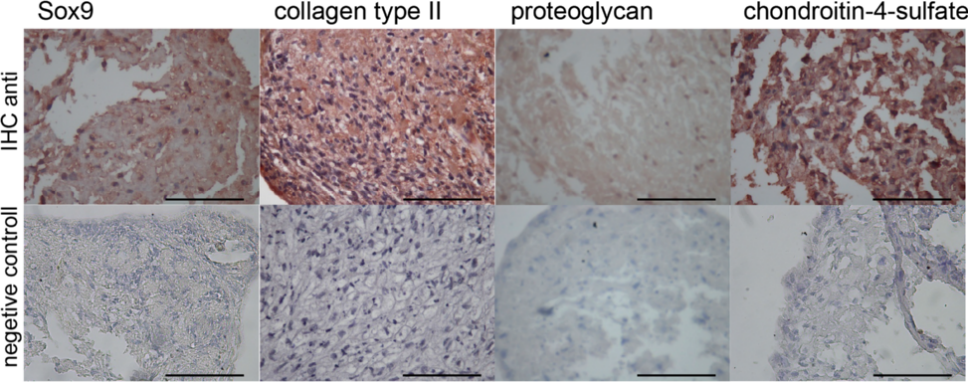
Chondrogenic differentiation of mononuclear cells isolated after AAP. High density culture after 21 days were stained against chondrogenic markers: Sox9, collagen II, proteoglycan, chondroitin-4-sulfate. *Second lane* shows no-primary controls. *Bar* represents 100 μm

Finally immunohistochemical stainings were performed on isolated cells of postoperative joint effusions growing in high density cultures (Fig. 4). After 21 days of culture, the cells showed strong positive staining for Sox9, Col II, proteoglycan and chondroitin-4-sulfate indicating the chondrogenic differentiation.

## Discussion

In this study we provide evidence for a biological mechanism that can in part explain the effectiveness of the common AAP procedure. We hypothesized that our findings have long been assumed by orthopedic surgeons who regularly indicate AAP as a treatment option without a clear scientific explanation.

Postoperative joint effusions as such have repeatedly given rise to extensive discussion about whether or not to use suction drainage [13]. Although many arthroscopic surgeons favour the use of wound suction drains, clear clinical evidence of their effectiveness is still missing. A limited number of studies do indicate that the effect of hemarthosis is greater during the first post-operational weeks if no intra-articular drain is inserted, but no differences in clinical outcome in terms of pain, infections and range of motion could be detected in short- and long-term follow-ups [13, 14]. We could detect a significant increase in the chondrogenic growthfactors TGFβ and IGF compared to serum level. Also the BMP4 levels is increased compared to normal serum level in literature (BMP4 serum level: 17.8 ± 35.2) [15] while the serum level of the osteogenic growth factor BMP7 is at the same level after AAP and ACP compared to serum level in literature (BMP7 serum level: 27 ± 82.2 pg/ml) [15]. Our study reveals the presence of a powerful mixture of MSC and chondrogenic growth factors in joint effusions. It appears thus questionable whether or not wound drainage is beneficial after arthroscopic procedures or even counterproductive, as a considerable amount of regenerative potential may be drained out of the joint. Our results do rather imply not to use postoperative drainage in order to preserve the healing potential of hemarthrosis and joint effusion after AAP.

Clearly, postoperative joint effusions consist of remnants of irrigation fluid and hemarthrosis as well as any tissue not irrigated. We here compared the content of mononuclear cells in effusions after AAP and ACP demonstrating that despite some dilution by irrigation fluid the cellular content is by far higher after the marrow-stimulating procedure.

Our findings are supported by the molecular analysis of surface proteins that clearly identify cells preserved from postoperative effusions, especially MSC. Flow cytometry is well recognized as a standard detection method to identify cellular fractions within a sample. Cells presenting a CD73^+^, CD 90^+^, CD44^+^, CD 105^+^, CD34^−^ immunphenotype have shown typical properties of MSC as they can be used for culture initiation and are able to form secondary colonies [12, 16]. Cells exhibiting such properties even after a 21 days culture of primary cells from joint effusions may be the actor of a powerful repair mechanism that can be initiated by AAP.

The cells released after AAP provide a chondrogenic phenotype in 3D cultivation. We were able to detect the classical chondrogenic differentiation marker Sox9, Col II, proteoglycan and chondroitin-4-sulfate by immunostaining. Unfortunately there was no possibility to perform high density culturing of ACP gained cells. The number of ACP gained cells was to low after 3 weeks expansion. A further increase of the expansion phase would lead to an aging process of the mononuclear cells which make a direct comparison between AAC and AAP difficult.

Chondrogenic differentiation capacity has long been reported for MSC and subsequently Sox9 was identified to be a master regulator of chondrogenic differentiation [17–19]. Therefore in order to improve cartilage repair modern concepts involve autologous MSC delivery into chondral defects [20]. Recent reports on intra-articular injection of MSC do imply that MSC within a joint effusion can integrate into cartilage lesions. Hence one proof-of-concept study demonstrates clinical improvement of patients suffering from painful osteoarthritis of the knee [10].

Chondrogenic differentiation is promoted by specific growth factors and cytokines of which we analyzed IGF-1 and TGF-β (for a review see [21]). BMP-4 and BMP-7 contents were also measured in the cell-free fraction of the joint effusions as they play a pivotal role in MSC-derived chondrogenesis [22, 23]. Notably, there were no significant differences between chondroplasty alone and AAP, whereas the levels of IGF-1, TGF-β and BMP-4 were markedly higher than serum levels. Possibly the release of these growth factors is triggered by the surgical intervention itself. These factors are able to act synergistically with a high number of other regenerative cells.

Abrasion arthroplasty is used for cartilage lesions of any grade. Typically Outerbridge grade II-IV lesions do become clinically symptomatic and lead surgeons to therapeutic action [7, 24]. Here, we are concentrating on AAP because it is easy to perform especially if cartilage lesions are seen during a diagnostic procedure. For most other marrow-stimulating procedures, special drills have to be used whereas AAP can be performed with a shaver. The Procedure involves debridement of all loose chondral tissue, scar tissue and subchondral bone to achieve access to cancellous bone. As such microfracture might yield comparable results [1, 3, 25].

Our study has some inherent limitations. Depending on the grade of osteoarthritic leasions the surgeon has choosen the operation technique in order to preserve healthy cartilage as much as possible. This led to an imbalance between the two investigated collectives. An aged matched analysis would further strengthen our conclusions.

Also the number of MSCs in the synovial fluid decreases with progressing osteoarthritic we were able to detect MSCs like cells in joint effusions after AAP also the average age of the patients were higher with a higher outerbrige score [8].

## Conclusion

In summary, we are providing novel scientific evidence for the biological effectivity of a marrow-stimulating arthroscopic procedure. This report supports the clinical application of AAP and furthermore provides some arguments against the use of postoperative suction drainage.

## Abbreviations

AAP: Abrasion Arthroplasty
ACP: Abrasion Chondroplasty
BMP: Bone morphogenetic protein
CD: Cluster of differentiation
Col II: Collagen type II
IGF-1: Insulin like growth factor
MSC: Mesenchymal stem cells
TGF beta: Transforming growth factor beta
